# Deformation Behavior of Bulk Metallic Glasses and High Entropy Alloys under Complex Stress Fields: A Review

**DOI:** 10.3390/e21010054

**Published:** 2019-01-11

**Authors:** Shunhua Chen, Jingyuan Wang, Lei Xia, Yucheng Wu

**Affiliations:** 1 School of Mechanical Engineering, Hefei University of Technology, Hefei 230009, China; 2 National-Local Joint Engineering Research Centre of Nonferrous Metals and Processing Technology, Hefei 230009, China; 3 Laboratory for Microstructures, Shanghai University, Shanghai 200444, China; 4 School of Materials Science and Engineering, Hefei University of Technology, Hefei 230009, China

**Keywords:** bulk metallic glass, complex stress field, shear band, flow serration, deformation mechanism, high entropy alloy

## Abstract

The plastic deformation of bulk metallic glasses (BMGs) depends significantly on applied stress states, and more importantly, in practical applications of BMGs as structural materials, they always deform under complex stress fields. The understanding of deformation behavior of BMGs under complex stress fields is important not only for uncovering the plastic deformation mechanisms of BMGs, but also for developing BMG components with excellent mechanical performance. In this article, we briefly summarize the recent research progress on the deformation behavior of BMGs under complex stress fields, including the formation and propagation of shear bands, tunable macroscopic plasticity, and serrated plastic flows. The effect of complex stress fields on the plastic deformation mechanisms of BMGs is discussed from simple stress gradient to tailored complex stress fields. The deformation behavior of high entropy alloys (HEAs) under complex stress states has also been discussed. Challenges, potential implications and some unresolved issues are proposed.

## 1. Introduction

As a new class of structural materials, bulk metallic glasses (BMGs) are considered poised for widespread engineering applications [1]. With non-ordered atomic structures, plastic deformation in BMGs is accommodated by initiation and propagation of shear bands, rather than dislocation movements as in crystalline alloys [2,3,4]. Under applied stress, plastic strain is localized within a thin layer of atoms with a width of about 2–210 nm, forming a shear band [5,6,7,8,9,10]. Extensive studies have shown that the initiation and propagation of shear bands are significantly dependent on applied stress fields. For example, the burst of a shear band relies on applied shear stress, and a shear band can be initiated only when the applied shear stress exceeds a critical value [5,11,12,13,14]. In addition, in practical applications of BMGs as structural materials, they always deform under complex stress states [15]. Studies have shown that BMGs can demonstrate more plastic deformation under complex stress states [16,17,18]. This implies that although some BMGs are brittle under conventional compression/tension tests with relatively-uniform stress fields, they can still have large capability to deform plastically in practical structural applications. For example, BMG foams can exhibit large nominal plasticity, which is much larger than solid BMG specimens [19,20,21]. BMG honeycombs and cellular BMG structures also demonstrate similar behavior [22,23,24]. The understanding of the deformation behavior of BMGs under complex stress fields is therefore important not only for uncovering the plastic deformation mechanisms of BMGs, but also for developing BMG components with excellent mechanical performance. Although many papers have reviewed the atomic structures, mechanical and physical properties, and functional and structural applications of BMGs [2,3,4,25,26,27,28,29,30,31,32,33,34,35,36,37,38,39,40], a comprehensive review on the plastic deformation behavior of BMGs under complex stress fields has not been reported. In this work, the research progress on the plastic deformation behavior of BMGs under complex stress fields is reviewed, including the initiation and propagation of shear bands, macroscopic plastic deformation behavior, criticality of plastic flows, and transition of deformation modes. The recent contributions on the deformation behavior of high entropy alloys (HEAs) under complex stress fields have also been discussed. The challenges, unresolved issues as well as future research directions are proposed.

## 2. Initiation of Shear Bands under Complex Stress Fields

Driven by applied shear stress, the dilatancy of atoms, associated with excess free volume, results in the decrease of viscosity localized in thin layers of shear bands [41,42]. The localization process causes local heating/softening, which was evidenced by experimental observations through fusible coatings [43,44,45]. The initiation of shear bands in BMGs was studied extensively during the past decades. Greer et al. [4] have summarized the formation mechanisms into three scenarios: the percolation of homogeneous nucleated shear transformation zones (STZs), stemming from the intrinsic structural fluctuations; the nucleation from the sites with extrinsically introduced stress concentrators, such as casting defects; and a two-consecutive-stage formation, including the burst of a viable band from activated STZs at stress-concentrated sites and a following rapid sliding process. Although the shear-banding mechanisms in BMGs are still under debate, all three kinds of scenarios share a common point that the initiation of shear bands is significantly dependent on applied stress fields. Johnson and Samwer [46] have shown that the potential barrier for activating STZs at a given shear stress, *τ*, can be expressed as *W*_τ_ = 4*RG*_0_*γ*_C_^2^[(*τ*_C_−*τ*)/*τ*_C_]^3/2^*ζ*Ω, where *τ*_C_ is the critical shear stress of the BMG, *γ*_C_ is the critical shear strain, *R* is a constant parameter, *G*_0_ is the shear modulus of unstressed BMGs at 0 K, Ω is the volume of STZs, and *ζ* is a correction factor. With the change of applied shear stress (*τ*) under complex stress fields, the activation process of STZs will be varied accordingly, resulting in differences in the initiation of shear bands. On the other hand, the annealing of stressed MGs can be used to shape MG films without embrittlement, where the change of free volume in stressed BMGs may also cause different deformation behavior [47]. Hufnagel et al. [48] have indicated that although many previous studies have focused on the deformation behavior of BMGs under uniaxial stress states, the understanding of the deformation behavior as well as structural evolution under more complex stress states is urgently needed to uncover the shear-banding mechanisms, such as the shear localization process. The research progress on the formation mechanisms of shear bands in BMGs has been summarized in many review papers [4,27,30,34,35,48,49,50,51,52,53,54], however, the formation of shear bands under complex stress fields has rarely been emphasized. Here, the initiation of shear bands under more complex stress fields is summarized.

**Figure 1:**
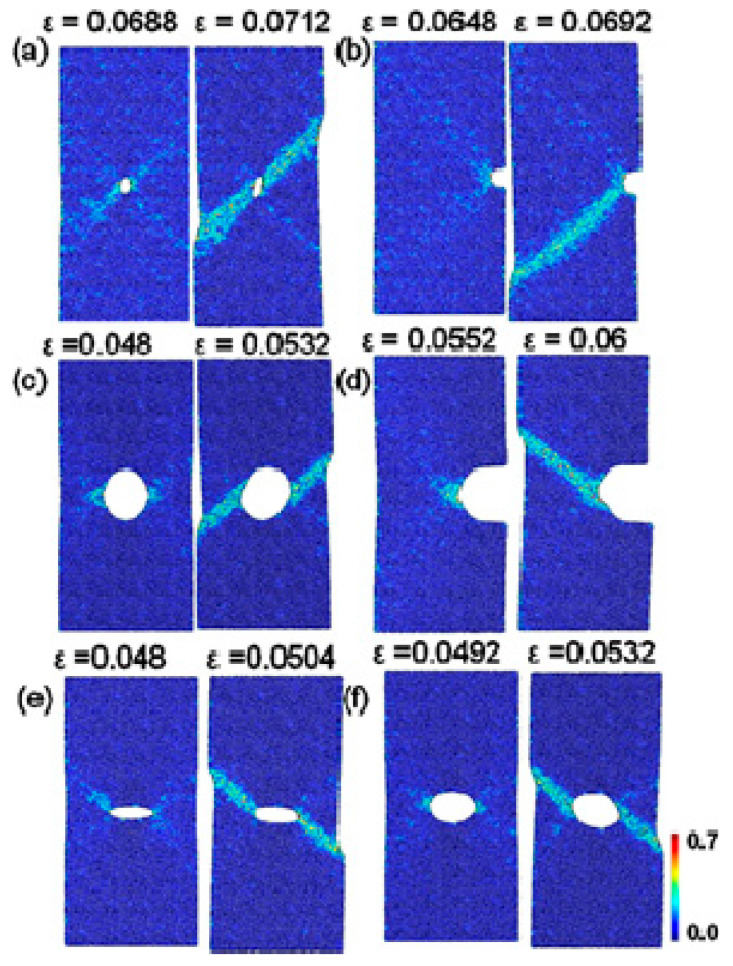
MD simulation results showing the initiation and propagation of shear bands in some notched Cu_50_Zr_50_ MG specimens, where the color denotes the localized shear strains. Reprinted from [60] with permission of 2013 AIP Publishing LLC.

With well-known size effect, MGs demonstrate different deformation behavior at submicron scales, for example, larger elastic limit, higher ductility, and more homogeneously plastic deformation with necking effect [55,56,57,58,59]. The introducing of stress concentrators in submicron-sized specimens will facilitate the initiation of shear bands, giving better insight into the shear band formation mechanisms. A simple method to introduce stress concentrators is to create notches in testing specimens. Based on MD simulations of some notched CuZr MGs, Sha et al. [60] have reported that shear bands emanate from the notch root when the plastic zone size ahead of the notches increases beyond a critical value. As shown in Figure 1, the notches can serve as stress concentrators to facilitate the localization of plastic zones, leading to the initiation of shear bands. Following studies have examined the effect of notch geometries and sizes on the formation of shear bands [61,62,63]. They have shown that the initiation of shear bands can be tailored by introducing notches with appropriate geometries and sizes, which can serve as stress concentrators for highly localized shear strains. By creating surface roughness in MG nano-pillars can also achieve similar localized shear strains [64]. It can then be concluded that under complex stress fields, stress concentrators serve as origin sites for the initiation of shear bands due to higher localized shear strains. The authors would like to point out that at sub-micron scales, the formation of localized shear bands can also be suppressed till fracture due to the change of deformation modes [65]. This phenomenon has also been observed in notched MG specimens with sub-micron sizes, where the change of notch sizes can tune the deformation modes from localized shear-banding to homogeneous softening and necking [61,62,63]. A summary on the homogeneous deformation behavior of BMGs under complex stress fields will be given in Section 5 in this paper, and thus not described in detail here.

Despite the efforts to understand the initiation of shear bands in BMGs under complex stress fields, some problems are still unresolved. For example, how to control the propagation of shear bands through the design of complex stress distributions? With well-known size effect, what’s the difference between the mechanisms on the formation of shear bands at submicron scales and macroscopic scales? Further understandings of the initiation and propagation of shear bands under complex stress fields could add more knowledge to the plastic deformation mechanisms of BMGs.

## 3. Macroscopic Deformation Behavior under Gradient Stress Distribution

The macroscopic plastic deformation in BMGs is accommodated by the initiation and propagation of shear bands. The rapid propagation of shear bands causes catastrophic failures, while the confinement of the propagation of shear bands can enhance the macroscopic plasticity. Moreover, the impeding of the propagation of shear bands is also beneficial for the initiation and bifurcation of more shear bands, leading to more plastic deformation. The applied complex stress fields in BMGs not only bring to stress concentrators, serving as sites for the initiation of shear bands, but also influence the propagation of shear bands, even under simple gradient stress distributions. Studies on the deformation behavior of BMGs under gradient stress distributions, for example, the specimens with tailored sample geometries and surface treatments, are discussed here.

### 3.1. Stress Gradient Resulting from Tailored Sample Geometry (Loading Angle)

**Figure 2:**
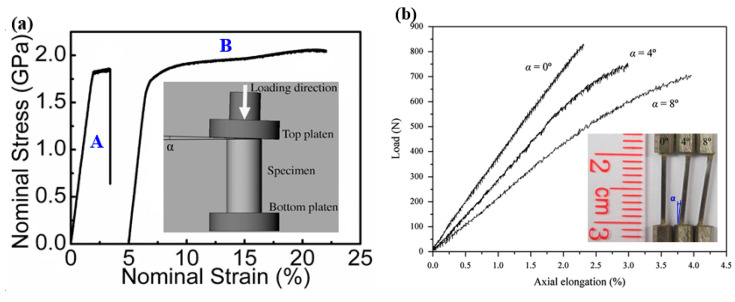
(**a**) Effect of stress gradient on the compressive plastic deformation behavior of BMGs, where the gradient stress distribution was introduced by tailoring a loading angle between the plateau and the specimen surface. A and B are macroscopic nominal stress-strain curves of the specimens without and with stress gradient, respectively (adapted from [16] with permission of 2008 AIP Publishing LLC.); (**b**) Effect of stress gradient on the deformation behavior of BMGs under tensile loadings, where the gradient stress distribution was introduced by designing Z-shaped specimens (adapted from [70] with permission of Elsevier).

Under compression tests of BMGs, by tailoring a tilted angle between the end surface and loading platen, as shown in Figure 2a inset, significant improvement in the macroscopic plasticity, associated with work-hardening-like behavior, was observed [16]. Based on the scanning electron microscopy (SEM) observations and Finite Element Modeling (FEM) results, Chen et al. [16] have shown that the propagation of shear bands can be stopped in the regions with relatively lower stress concentration orders, leading to the multiplication of more shear bands associated with greatly enhanced macroscopic plasticity. Similar large nominal plasticity was also observed in some other BMG samples with tilted surface angles [17,66,67]. The tilted geometry constrained the initiation of shear bands in the regions with localized strains, and at the same time hindered the propagation of shear bands, leading to the multiplication of more shear bands. Despite the brittleness, some BMG communities still have certain plasticity under compression tests. However, almost all communities of BMGs are brittle under tensile loadings. The investigations on the plastic deformation behavior of the tensile side of bending BMG specimens have indicated that more plastic deformation was found due to the presence of tensile stress gradient [68,69]. The gradient stress distributions may also be possible to be used to improve the plastic deformation behavior of BMGs under tensile loadings. Chen et al. [70] have then introduced gradient stress distribution into some tensile specimens by tailoring tilted angles in Z-shaped BMG specimens, as shown in Figure 2b. It is interesting to find that the tilted BMG specimens have demonstrated more plastic deformation in the stress-concentrated regions, similar to compressive testing results. This implies that although BMGs are brittle in conventional compression/tension tests with relatively uniform stress distributions, they may still demonstrate more plastic deformation in practical engineering applications, where they always deform under complex stress fields. Additionally, with the presence of stress gradients, BMGs can also exhibit attractive performance for engineering applications, such as higher reliability in nominal strains [71] and less dependence on the change of loading rates [72]. The understanding of the deformation behavior of BMGs under stress gradients opens a new window for the practical applications of BMGs.

### 3.2. Effect of Surface Residual Stress

**Figure 3:**
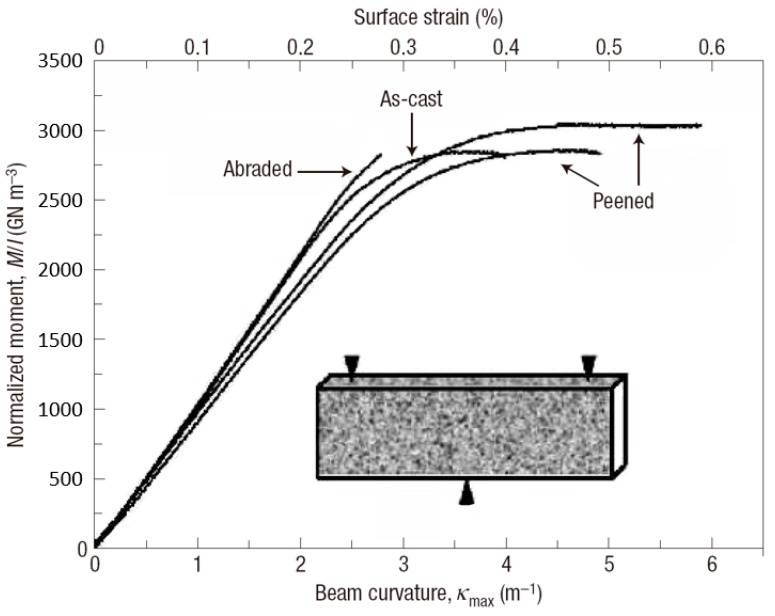
Three-point bending behavior of BMGs with surface treatment by shot peening. Reprinted from [73] with permission of Springer Nature.

In engineering applications of structural materials, residual stress is usually created to improve the mechanical performance. In BMG communities, the introducing of surface residual stress using techniques, such as shot peening, laser melting and mechanical attrition, has also been investigated to improve the macroscopic plastic deformation behavior. For example, Zhang et al. [73] have treated some Zr-based BMGs using shot peening, and enhanced the bending plasticity significantly (Figure 3). A maximum surface plastic strain of about 0.35% was obtained while the as-cast BMG specimen only has a limited value of about 0.15%. They have shown that the compressive residual stress on the surface of shot-peened specimens can suppress the propagation of shear bands and cracks. Combined with the pre-existed shear bands nucleated underneath the surface, more shear bands will be initiated to achieve larger macroscopic plasticity. By laser surface melting, gradient distribution of both tensile and compressive residual stresses as well as atomic structural changes can also be employed to improve the macroscopic plasticity of BMGs [74,75]. In addition, Wang et al. [76] have obtained an obvious enhancement in the tensile plasticity of BMGs by surface mechanical attrition treatment. Gradient atomic structures were formed during the mechanical attrition process, resulting in a gradient distribution of residual stress with both compressive and tensile stresses. They have shown that the gradient distribution of residual stress can cause the formation of more shear bands, and at the same time, delay the cavitation effect, resulting in a work-hardening mechanism associated with enhanced plasticity [76].

Despite the use of different methods/techniques to introduce gradient stress distributions to BMGs, it can be concluded that the localized regions with high orders of stress concentration can promote the initiation of more shear bands, and the regions with less stress concentrations impede their propagations. This will further induce the bifurcation and multiplication of shear bands, leading to better macroscopic plasticity. By design of appropriate distributions of more complex stress fields, it is able to tailor the formation and propagation of shear bands, achieving controllable plastic deformation behavior. In fact, many studies have been devoted to achieving tunable plastic deformation behavior of BMGs by designing complex stress fields, which are given in the following section.

## 4. Tunable Plastic Deformation Behavior under Tailored Complex Stress Fields

During the past decade, tunable plastic deformation behavior has been widely achieved by extensive studies based on the design of complex stress fields, for example, to guide the propagation of shear bands and cracks, to tune the criticality of the plastic-flow dynamics, and to obtain large apparent plasticity/elongations. The studies are of significance for giving more insight into the shear-banding mechanisms of BMGs, and obtaining better macroscopic plastic deformation performance for engineering applications.

### 4.1. Guiding the Propagation of Shear Bands and Cracks

The initiation of shear bands emanating from notch roots was widely studied to evaluate the fracture behavior of BMGs [77,78]. Tandaiya et al. have shown that by changing the mixity of loading modes (I/II) of bending specimens can tune the plastic zone sizes as well as their distributions [79,80].

**Figure 4:**
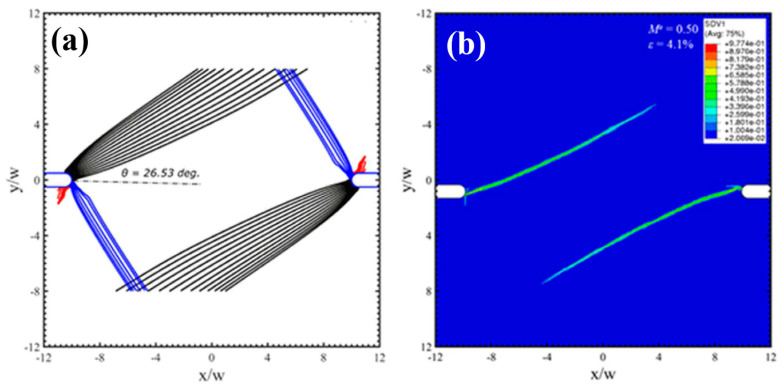

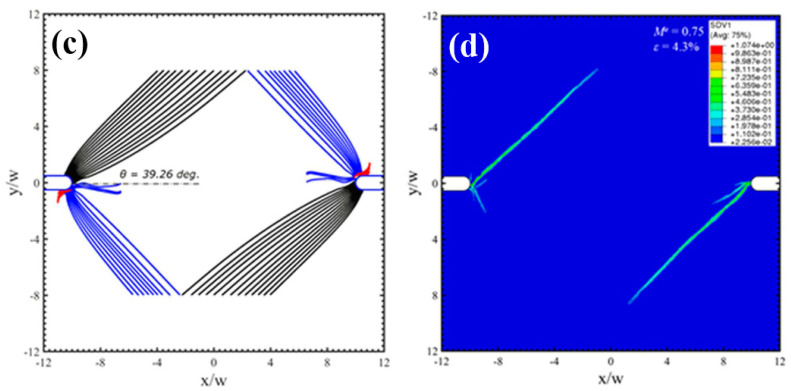
Shear bands formation in notched specimens with mode mixity (M^e^) of 0.5 (**a**,**b**) and 0.75 (**c**,**d**), respectively (M^e^ = 1 and 0 stand for mode-I and mode-II loading conditions, respectively). (**a**,**c**) show the predictions by an instability theory, and (**b**,**d**) are the FEM results [83].

Under mixed mode loading conditions, the notch root deforms as that one part sharpens and the other part blunts. The increase of mode II component can enlarge the plastic zone sizes and enhance the localized strain levels ahead of the notch tips, resulting in controllable directions for shear-banding and micro cracks. For some notched BMGs, Yi et al. [81] have demonstrated that by creating pre-existed shear bands can change the plastic deformation behavior as well as the crack propagations around the notch root. To guide and deflect the cracks in those notched BMGs can improve the fracture resistance behavior, which is useful for toughening BMG components with stress concentrations in engineering applications. The controlled shear-banding behavior of notched BMGs was understood by Yang et al. from a perspective of “multiple shear band deformation mechanisms”, differing from the conventional materials fracture mechanics [82]. The proposed fracture mechanisms agree well the fracture morphologies of notched BMGs, giving more theoretical understandings on the controlling of shear-banding in notched BMGs. Li et al. [83] have also conducted theoretical analysis on the controlling of shear-banding in BMGs using an instability theory. As shown in Figure 4, the formation of shear bands in notched specimens with mode mixities of 0.5 and 0.75 was successfully predicted by instability analysis, which is highly in line with the FEM results and experimental observations [83]. Such a theory may be further used to predict the shear-banding behavior of BMGs under complex stress fields in practical engineering applications.

### 4.2. Tunable Criticality in Flow Serrations

**Figure 5:**
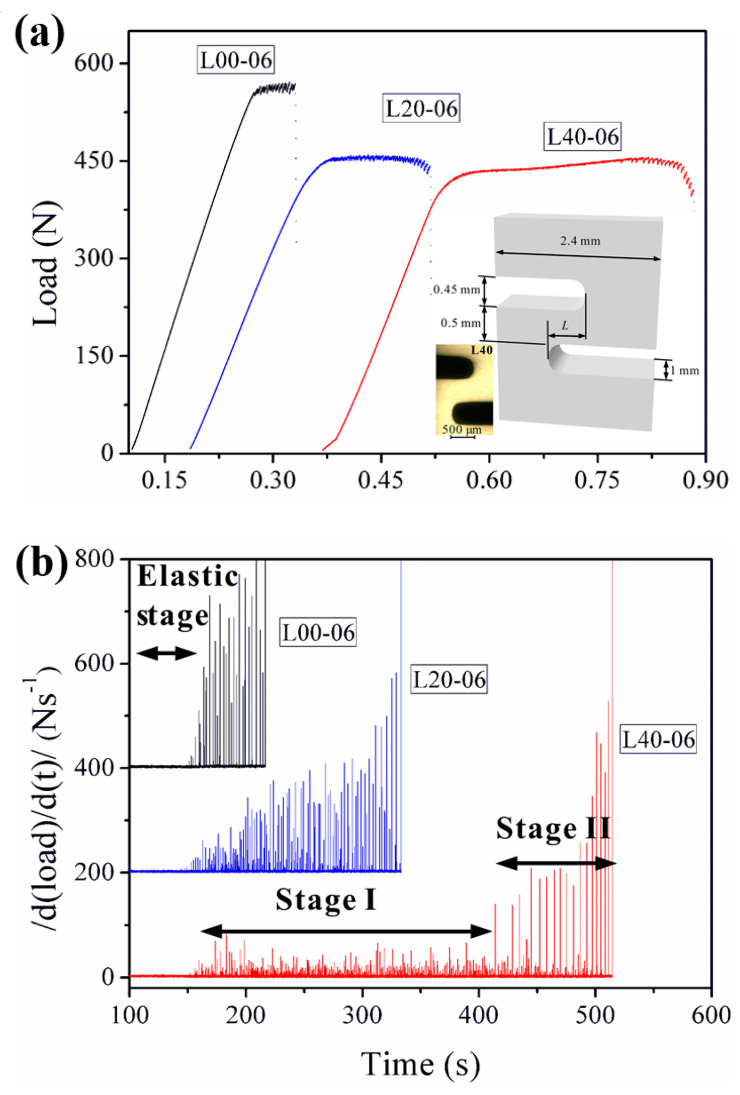
(**a**) Serrated plastic flows of three notched BMG specimens at a loading rate of 0.06 mm/min., where L = 0, 0.2, and 0.4 mm for L00, L20 and L40 specimens, respectively. (**b**) The |d(load)/d(t)| versus time relationships showing the observation of two-stage plastic flows [96].

The discontinuous plastic flows in crystalline alloys display serrations or jerky flows in stress-strain curves, which are known as the Portevin-Le Chatelier (PLC) effect [84,85]. Although BMGs have vanished crystalline lattices, the plastic deformation in BMGs is limited to inhomogeneously-localized shear bands, which also results in discontinuous stress-strain behavior similar to the PLC effect in crystalline alloys, i.e., serrated plastic flows. A great consideration of research has shown that the serrated plastic flows of BMGs can evolve to a power-law scaling criticality [40,86,87,88,89,90,91,92,93,94]. The criticality of the plastic-flow dynamics is usually correlated to larger macroscopic plasticity [86]. The criticality of the flow serrations can be attributed to the multiplication and intersections of shear bands, which suggests that the burst of shear bands may be intrinsically correlated [86,95]. However, for BMGs under tensile loadings, the catastrophic failures lead to limited serrations where the understanding of the plastic-flow dynamics is very challenging. By designing complex stress fields through double-side notches, Chen et al. [96] have reported tunable criticality in the plastic flows of BMGs under mixed mode (I/II) loading conditions. As shown in Figure 5, by the multiplication of shear bands within the regions with complex stress fields, a stable plastic flow stage was observed, resulting in the delay of catastrophic failures [96]. The two-stage plastic flows were also observed under compression tests, where the formation of new shear bands tends to delay catastrophic failures [97]. For the notched specimens with complex stress fields (Figure 5), the flow serrations during the stable plastic flow stages have smaller magnitudes, and evolve to a power-law scaling. The findings suggest that it may be possible to obtain more flow serrations in tensile BMGs by introducing of complex stress fields, where enough serration data could be collected to give more insight into the plastic-flow dynamics of BMGs under tensile loadings.

Thereafter, by tailoring single-side notches with varying radii, complex stress fields with varying stress concentration factors have been introduced to some tensile BMG specimens [76]. Despite different trends in the evolution of amplitudes from compression tests, the flow serrations of the single-side notched BMG specimens also demonstrate power-law criticality within the stable plastic flow stages, similar to the results under compression and nano-indentation tests [76]. The power-law criticality in BMGs might be a universal rule for all kinds of loading conditions. Based on such assumption, the catastrophic failures in BMGs can then be delayed or avoided in practical applications by designing complex stress fields, regardless of loading conditions. Additionally, under complex stress fields, BMGs can also have high uniformity for accumulating elastic energy during the stress-arising process of the flow serrations [98]. By tailoring of complex stress fields is helpful for uncovering the mechanisms of the plastic flow serrations in BMGs and to achieve better plastic performance.

### 4.3. Achieving Large Macroscopic Plasticity/Axial Elongation

**Figure 6:**
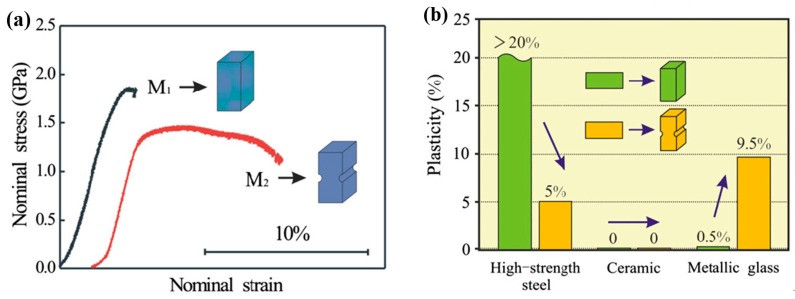
Enhanced nominal strain in a notched BMG specimen under compression (**a**), and the comparison of the effect in different materials (**b**). Adapted from [102] with permission of Elsevier.

The improved macroscopic plastic deformation behavior of BMGs under complex stress fields can be observed in some specimens with casting defects, such as voids [99]. In order to create complex stress fields to block the propagation of shear bands, Zhao et al. [100,101,102,103] have introduced notches into BMGs and obtained greatly enhanced nominal strains. An example showing the improvement of nominal strains of notched BMGs was shown in Figure 6a. The introducing of symmetrical notches can result in stable plastic flows confined within the regions between notches. With high orders of stress concentrations, shear bands are easier initiated from the notch bottoms. However, only the stress fields between two symmetrical notches can effectively confine the propagation of shear bands. In the specimens with single notches or two asymmetric notches, significant improvement in nominal plasticity was not observed due to the rapid propagation of shear bands [100,101,102,103]. By tailoring the distribution of more notches is useful for achieving larger macroscopic plasticity of BMGs, especially in practical applications of BMGs [104]. Moreover, such a strategy may be particular useful for BMGs due to the unique atomic orders. The notched high-strength steel and ceramic specimens exhibited no obvious improvement or even decreased nominal strains (Figure 6b) [102].

**Figure 7:**
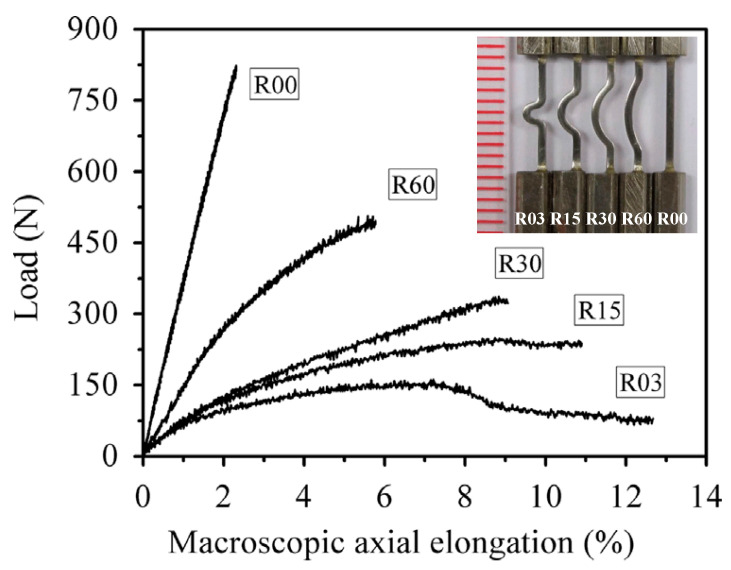
Tunable large axial elongations in curved BMG specimens under tensile loadings, where the inset image showing the reduced sections of the curved specimens. Adapted from [108] with permission of Elsevier.

As compared with the plastic deformation behavior of BMGs under compressive loadings, it is challenging to obtain plasticity under tensile loadings due to catastrophic failures. With vanishing of crystalline lattices and boundaries, the rapid propagation of shear bands cannot be impeded, and leads to brittle failures. Nevertheless, it is fortunately to find that the introducing of complex stress fields can be employed to improve the plastic deformation behavior of the stress-concentrated regions. Qu et al. [105,106] have reported that the complex stress field created by double-side notches can prevent the unstable propagation of shear bands, and result in a stable plastic zone. Li et al. [107] have further examined the effect of notch sizes and shapes on the formation and propagation of shear bands in the stress-concentrated regions, based on FEM simulations, and identified the notch conditions which are beneficial for plastic deformation. By tailoring the plastic deformation in stress-concentrated regions, tunable axial elongations have also been obtained in some curved specimens, as shown in Figure 7 [108]. With both compressive and tensile stresses, the curved segment forms a shear band multiplication stage during tensile loading process [18,108]. Although the large plastic deformation was localized in the stress-concentrated regions, the straightening of the curved segments results in large axial elongations along the loading directions. Despite the brittleness under uniform stress distributions, large axial elongations can still be achieved in BMGs and BMG structures through the geometry design, laying a sound foundation for the practical applications of BMGs as structural materials.

Additionally, tensile ductility in BMGs has also been achieved by introducing complex stress states onto the surface of BMG specimens, such as imprinting method [109,110] and laser surface treatment [111,112]. The enhancement of macroscopic tensile ductility was attributed to the heterogeneities on the specimen surface, such as the mechanical properties and geometries, resulting from tailored complex stress fields [109,110]. The heterogeneities cause the formation of plastic zones with multiple shear bands. The multiplication and intersection of shear bands limit the rapid propagation of shear bands, and therefore enhance the macroscopic ductility. Gao et al. [111] have found that with complex stress fields introduced by laser surface treatment, the shear band propagation was impeded, leading to a relatively homogeneous deformation mode. Dong et al. [112] have further shown that the tensile ductility may be ascribed to the large scale flow, driven by the complex stress fields introduced by laser engraving. Although the mechanisms on the enhancement of macroscopic ductility of BMGs under complex stress fields are still being debated, in fact, complex stress fields have been widely employed to investigate the deformation mechanisms of BMGs, i.e., under indentation tests. For example, indentation tests have been used to investigate the shear band formation and propagation mechanisms [113,114], and the transition of deformation modes from serrated flows to homogeneous deformation [115,116]. However, since the specimens do not fracture during indentation tests and the studies mainly focused on the corresponding deformation mechanisms, these studies on the indentation tests of BMGs are therefore not discussed in detail here.

Up to date, complex stress fields have been used to characterize the shear band formation and propagation mechanisms, as well as the global deformation behavior, such as the plasticity and fracture. However, theories on how to control the propagation of shear bands and cracks, and subsequently control the macroscopic plastic deformation behavior of BMGs are still urgently needed. In the practical applications of BMGs as structural materials, most of the parts of BMG structures may deform under complex stress fields. The understanding of the macroscopic deformation behavior of BMGs under complex stress fields is also useful for designing BMG structures with better mechanical performance, for example, the development of BMG foams [20], BMG honeycombs [22] and cellular BMGs [117].

## 5. Transition of Deformation Modes under Complex Stress Fields

**Figure 8:**
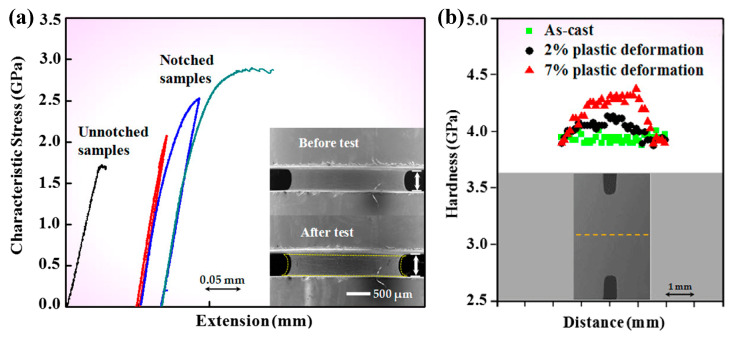
(**a**) Strain hardening effect in a notched BMG at room temperature, and (**b**) shows the trace of mircohardness at the notched regions. Adapted from [119] with permission of American Physical Society.

Attributed to the amorphous atomic structures, homogeneous plastic flow in BMGs is usually observed at high temperature [42]. However, at room temperature, the introducing of complex stress fields can also result in the transition of deformation modes from highly localized shear-banding to relatively homogeneous deformation. A pioneered work by Flores and Dauskardte [118] on the strain localization behavior of some notched BMG bars has suggested that the stress states may affect the deformation and failure behavior of BMGs. Want et al. [119] have then examined the plastic deformation behavior of a Zr_64.13_Cu_15.75_Ni_10.12_Al_10_ (at. %) BMG under multi-axial tensile stress states, introduced by circumferential deep notches. It was surprising to find that the notched BMG specimens demonstrate strain hardening behavior at room temperature, as can be seen in Figure 8. This unusual phenomenon was attributed to the diffusional relaxation driven by multiaxial stress states, where obviously shear-banding behavior was not observed. Further studies have shown that the change of the notch dimensions can cause BMGs to deform plastically through the nucleation and coalescence of voids/cavies, where shear-banding behavior is suppressed [120]. Since the formation of shear bands occurs from very localized regions into sub-micron scales, the examination of homogeneous plastic deformation at sub-micron scales brings more understandings to the transition of deformation modes from localized shear-banding to homogeneous deformation.

**Figure 9:**
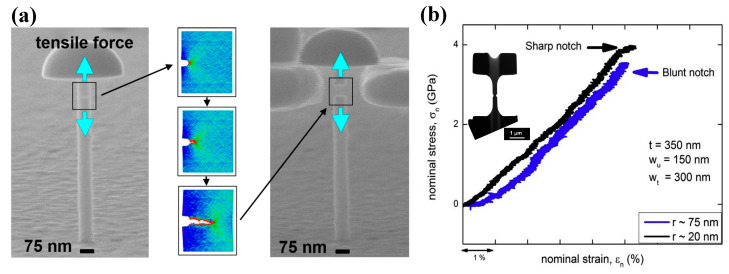
(**a**) Deformation behavior of single-side-notched Ni-P/Fe-P MGs (Reprinted from [121] with permission of ACS Publications); (**b**) in-situ TEM observation of the deformation behavior of double-side-notched CuZr MG specimens with varying sharpness (adapted from [122] with permission of Elsevier).

Gu et al. have examined the shear-banding and fracture behavior of some notched Ni-P/Fe-P MGs of about 70 nm in diameter, as shown in Figure 9a [121]. Combined with experimental observations and molecular dynamics (MD) simulations, they showed that plastic deformation in these notched MG specimens initiated from the notch root by forming microscopic voids, and the coalescence of voids resulted in cavitation and final brittle failures. Narayan et al. [122] have studied the deformation mechanisms of some double-side-notched CuZr MGs with varying sharpness (Figure 9b). They have found that the specimens with sharper notches can delay the formation of shear bands, resulting from a higher degree of triaxiality in stress distributions. When the cavitation stress reaches a threshold value, plastic deformation in MGs can transit from shear-banding to microscopic voids coalescence, similar to the MD simulation results reported by Gu et al. [121]. On the other hand, Narayan et al. [122] have also examined the effect of notch depth on the plastic deformation mechanisms of MGs. It was shown that a deeper notch tends to facilitate homogeneously activated STZs and then suppress shear-banding. However, to date the experimental studies on the formation of STZs and shear bands in notched MG specimens are not sufficient enough to make a consensus conclusion on the formation and evolution mechanisms of STZs/shear bands. Since STZs only involve a small cluster of atoms, it is still challenging but necessary to investigate the initiation of STZs under complex stress fields directly through in-situ TEM observations, and how these STZs evolve to shear bands/cracks. MD simulations are therefore more feasible to be employed to investigate the formation of shear bands under complex stress fields.

**Figure 10:**
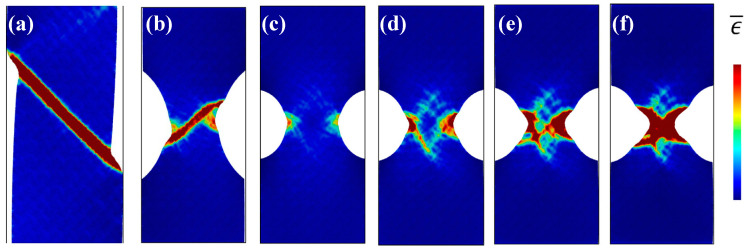
MD simulation results of the Mises strain plots of unnotched (**a**) and notched (**b**–**f**) specimens, where (**c**–**f**) shows the evolution of plastic zones in a specimen with a sharper notch than (**b**). Adapted from [62] with permission of Elsevier.

Based on MD simulations, many previous studies focused on the effect of notch sizes and geometries on the plastic deformation behavior of nanoscaled MG specimens, aiming to shed more light into the transition of deformation modes. Sha et al. have shown that the design of notches with increased depth and sharpness can suppress shear-banding and result in more homogeneous defamation with presence of necking [61]. Similar transition of deformation modes can also be observed in nanopillars with tailored surface roughness [64]. Pan et al. [123] have investigated the physical origin of the homogenous deformation, and found that voids and cavitations were initiated at the notch root when the stress triaxiality exceeds a critical value. On the other hand, Dutta et al. [62] have also observed the transition of deformation modes from shear-banding (Figure 10a,b) to homogeneous deformation with necking (Figure 10c,d). With a relatively sharper notch (Figure 10c), plastic zones first initiated from the notch root due to stress concentrations, and then evolved to incipient shear bands (Figure 10d). However, due to the rapid expansion and coalesce of plastic zones, the formation of shear bands was finally suppressed, showing a necking effect (Figure 10e,f). It is reasonable to see the initiation of some incipient shear bands, where stress concentrators can also serve as sites for the nucleation of STZs, as discussed in Section 2. There may exist a competing process for the formation of localized shear bands and the coalescence of plastic zones, where the rapid coalesce of plastic zones can suppress the initiation of shear bands and result in different plastic deformation mechanisms. This phenomenon is also highly in line with the in-situ TEM observations on the plastic deformation behavior of some nanosized MG specimens [122]. The change of atomic packing in a shear band was observed in a Ni-based MG [124]. More recently, Cui et al. [63] have examined the structural evolution of CuZr MGs during the transition of deformation modes from shear-banding to homogeneously necking in notched MGs. They have shown that the Voronoi volume recovery can be dominant in the localized regions with triaxial stress state, differing from the unnotched specimens. Nevertheless, more effort is still needed for revealing the underlying physical origins of the homogeneous plastic deformation within the localized regions with complex stress fields, such as the movements of atoms, atomic structural evolutions as well as the formation and coalesce of microscopic voids.

## 6. Deformation Behavior of HEAs under Complex Stress Fields

**Figure 11:**
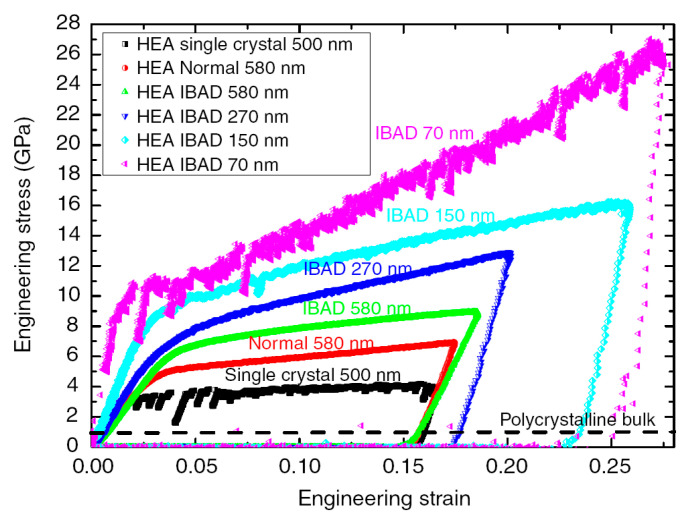
The compressive stress-strain curves of NbMoTaW HEAs, where IBAD stands for the specimens with Ar^+^ ion beam-assisted deposition [129].

HEAs are a new class of alloys, having at least five elements with equal or near equal atomic percentages, where the solvent and solute elements are not easily distinguished [125]. The mixing of multi component in the solution states results in very high entropy associated with unique properties. For example, they can have high strength comparable to BMGs [125]. More importantly, in conventional metallurgical methods, the increase of strength leads to the decrease of ductility, and it seems that the strength and ductility are usually mutually exclusive in conventional alloys [126]. However, some HEAs can exhibit both high strength and ductility [127]. The plastic deformation behavior of HEAs shares similar characteristics as compared with BMGs. For example, differing from conventional alloys, HEAs also have serrated plastic flows, resulting in challenges to accurately predict and control the plastic deformation behavior [40,128]. The serrated plastic flows of HEAs are also significantly dependent on the change of strain rates, temperature and sample dimensions [125,128,129]. For instance, Zou et al. [129] have examined the plastic deformation behavior of some HEAs with Ar+ ion beam-assisted deposition, where flow serrations were obviously observed in the pillar of 70 nm diameter, while the pillars with larger diameters tend to have smooth plastic flows (Figure 11).

Recent research has shown that the residual strains may cause the instability of phases and result in the transition of phases [130]. This may further change the mechanical properties of HEAs, for example, transition induced plasticity [127]. Joseph et al. [131] have reported that some laser-fabricated Al_0.3_CoCrFeNi HEAs exhibited tension/compression asymmetric deformation behavior, without/with mechanical twining. Therefore, the change of applied stress fields could also affect the evolution of microstructures in HEAs, such as the phase transition and mechanical twining, and the resultant flow serrations and mechanical properties. The tailoring of complex stress fields can be used to improve the plasticity and tune the criticality of plastic flows in BMGs, while some research has also shown that the notches can reduce the nominal strains of crystalline high-strength steels [102]. Whether the introducing of complex stress fields is helpful for improving the plasticity and tuning the criticality of flow serrations in HEAs is still a mystery. Up to date, a study focused on the deformation behavior of HEAs under complex stress fields has yet been reported. Regarding that the evolution of microstructures of HEAs is significantly affected by applied stress states, it should be worthy of further investigations on the deformation behavior/mechanisms of HEAs under complex stress fields.

## 7. Conclusions and Future Directions

The change of applied stress fields can significantly affect many aspects of the plastic deformation in BMGs, such as the shear band initiation and propagation, evolution of flow serration, and macroscopic plastic performance. Varying complex stress fields have been employed to investigate the mechanisms on the burst of shear bands, guiding the propagation of shear bands/cracks, tunable plastic-flow dynamics, transition of deformation modes and better plastic performance. On one hand, complex stress fields play a significant role for elucidating the mechanisms on the plastic deformation in BMGs. For example, how STZs are initiated and when the formation of STZs is suppressed? On the other hand, complex stress fields can be tailored to achieve controllable plastic deformation behavior for practical engineering applications. A case in point is to design BMG structures/devices with enhanced mechanical properties. However, the understanding on the deformation behavior of BMGs under complex stress fields still faces challenges, and some unresolved issues are summarized and given below:

- Although studies have been devoted to investigating the formation of shear bands under complex stress fields, how to control the formation of a shear band under a given complex stress fields is still challenging, especially under experimental observations. The localization of plastic deformation in BMGs at submicron scales involves size effect and transition of deformation modes. Due to different mechanical/physical properties, differences may exist during the formation of shear bands when characterized in specimens with varying sample dimensions. With complex stress fields, the formation and propagation of shear bands can be tailored, and even be eliminated by homogeneous deformation. The mechanisms on the plastic deformation of BMGs under complex stress fields are worthy of further attention to uncover the fundamental deformation/fracture mechanisms of BMGs.
- Extensive studies have shown that the burst of shear bands is not an independent event and affected by previously existing shear bands [73,96]. The flow serrations in BMGs, which are related to the formation of shear bands, may also have intrinsic links. Despite the well-known tunable power-law criticality, how to predict and control the serrated plastic flows in BMGs is still very difficult and challenging. The control of the initiation and propagation of shear bands under complex stress fields could be helpful for shedding more light into the underlying relationships among the bursts of flow serrations.
- The engineering applications of BMGs still have many challenges due to catastrophic failures and metastable microstructures, associated with uncertainty in mechanical properties. The achievement of controllable plastic deformation behavior under complex stress fields may not only improve the macroscopic mechanical performance, but also lead to more reliable behavior. Combining with tailored complex stress fields, BMG devices/structures with enhanced performance as well as predictable properties can be further developed, exploring the engineering applications of BMGs.
- HEAs have some characteristics similar to BMGs, for example, the serrated plastic flows which are difficult to predict/control. The introducing of complex stress fields can result in the evolution of microstructures, such as phase transition and mechanical twining, which may also be beneficial for uncovering the deformation mechanisms of HEAs. Furthermore, with controllable evolution of microstructures, the outstanding mechanical properties of HEAs could be further improved and optimized.
